# Hidden battles: dissecting host–fungal interactions and immune heterogeneity in infection outcomes

**DOI:** 10.1128/msphere.00873-25

**Published:** 2026-05-29

**Authors:** Rebecca A. Drummond

**Affiliations:** 1 Institute of Immunology and Immunotherapy, School of Infection, Inflammation and Immunity, College of Medical and Health Sciences, University of Birmingham 1724, Birmingham, United Kingdom; Virginia-Maryland College of Veterinary Medicine, Blacksburg, Virginia, USA

**Keywords:** fungi, meningitis, immune response, host-pathogen interactions, mycology

## Abstract

Fungal infections remain a major global human health problem, responsible for billions of infections and millions of deaths each year. Dissecting the immune mechanisms that protect us against these infections is an important step toward developing alternative therapies for patients at risk for fungal diseases, who are typically immunocompromised. Understanding antifungal immune responses is complicated by heterogeneity within host cell populations, as well as in-host adaptation strategies employed by fungi, which may also drive multiple distinct populations during infection. In this article, I discuss our recent discoveries and that of the medical mycology field, which has used creative and interdisciplinary approaches to better define the multiple layers of complexity that influence host–fungal interactions and infection outcomes. Finally, I describe examples where fundamental discovery has translated into new treatment approaches for patients and may represent revolutionary changes for our growing field.

## INTRODUCTION

When I began my research group in 2018, I was determined to use an interdisciplinary approach in our work, which focuses on the immunology of fungal meningitis and brain infections. In my original mSphere of Influence article, I discussed how reading widely outside my field was important for broadening perspective and developing new questions. This would still be one of my top recommendations to new principal investigators setting up their own groups. The interdisciplinary approach and collaborations we have set up over the past few years have been essential to our work, as our research has led us toward understanding the impact of host immune responses on microbiological phenotypes and the neurological consequences of infection. Here, I discuss what we have learned in the last 5 years and key new discoveries in medical mycology that have resulted from interdisciplinary ideas.

## HOST CELL HETEROGENEITY

Five years ago, I discussed work by neuroimmunologists, which described heterogeneity of tissue-resident macrophages in the brain, called microglia, for the first time. Microglia were found to develop into a phenotype called “damage-associated microglia” (DAMs) in the context of neurodegeneration and actively contribute toward pathology (1). Since this first report, DAMs have been described in other neurodegenerative disorders, and polymorphisms in DAM-associated genes have been identified in genome wide association study (GWAS) for these diseases, particularly Alzheimer’s (2). Curious as to whether microglia developed into DAMs or other functional subsets within the fungal-infected brain, my lab embarked on employing single-cell RNA sequencing in mouse models of cryptococcal meningitis to identify microglia and other myeloid cell functional subsets. We discovered that microglia responses typically occurred in the later phases of infection, when clinical symptoms of meningitis began to appear (3). At this point, we discovered a subset of microglia that highly expressed inflammatory markers and hallmarks of antimicrobial responses, yet these cells were poorly fungicidal and contributed toward pathologic inflammation in the brain (3). A similar observation was made by another lab using a different serotype of the fungus just a couple of months later (4). The microglia subset we discovered did not resemble DAMs, however. Instead, their transcriptional profile more closely matched that of microglia responding to systemic lipopolysaccharide (LPS) exposure (5). We therefore termed these microglia inflammation-associated microglia (IAMs) to match the nomenclature used in the LPS study, and we believe that these cells represent an activation state of microglia, which results from microbial stimulation.

**Fig 1:**
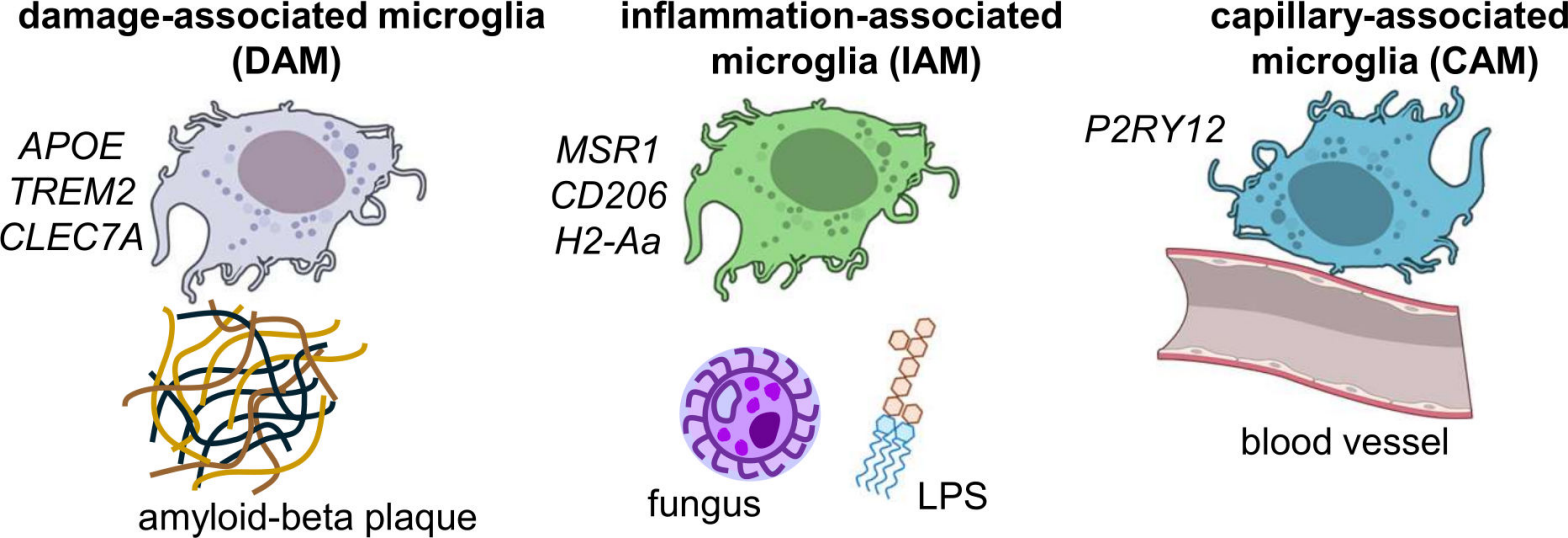
Microglia heterogeneity. The three main microglia activation states described in this article are shown, each with their main stimuli and key genetic markers. Macrophage and blood vessel icons are from the NIH BioArt repository.

Many other microglia subsets have since been described that are utilized in various cellular processes (Fig. 1). For example, detailed microscopy-based analyses helped identify a subset of microglia that reside alongside blood vessels and regulate blood flow and vasodilation in the brain (6). These capillary-associated microglia therefore performed an important homeostatic function within the central nervous system and were distinct from the more reactive microglia phenotypes that have been observed during neurodegeneration and infection (Fig. 1).

## FUNGAL ADAPTATION TO THE HOST

Our experiments on microglia heterogeneity and function revealed an additional layer of complexity: heterogeneous fungal populations. Fungi have been typically studied as one population from host tissues and clinical samples, yet these microbes can inhabit different niches with varying levels of nutrients and oxygen availability that will significantly affect their stress responses and host adaptation. *Cryptococcus neoformans*, the main causative agent of fungal meningitis in immunosuppressed humans, can either reside within large fungal communities called cryptococcomas in the brain or may survive intracellularly inside the phagosomes of myeloid cells, such as microglia (7). Recent work has also characterized new morphological phenotypes of *C. neoformans* that are better adapted for brain dissemination and have altered interactions with immune cells (8). We found that microglia harbored live *C. neoformans*, particularly the IAM population, but that intracellular yeast populations had different phenotypes compared to yeast residing extracellularly (the cryptococcomas) (3, 9). Extracellular yeast upregulate copper transporters to adapt to the low levels of available copper in the brain, and their ability to adapt to copper starvation is essential for their virulence (10). However, we found that *C. neoformans* residing intracellularly within microglia were protected from copper starvation, indicating that the yeast may be able to harvest copper from these host cells (9). This work led us to ask questions surrounding in-host adaptation strategies and how metabolic competition for resources fuels heterogeneity and influences host–fungal interactions. Indeed, this is an area within the medical mycology field that has seen several important advances in recent years.

**Fig 2:**
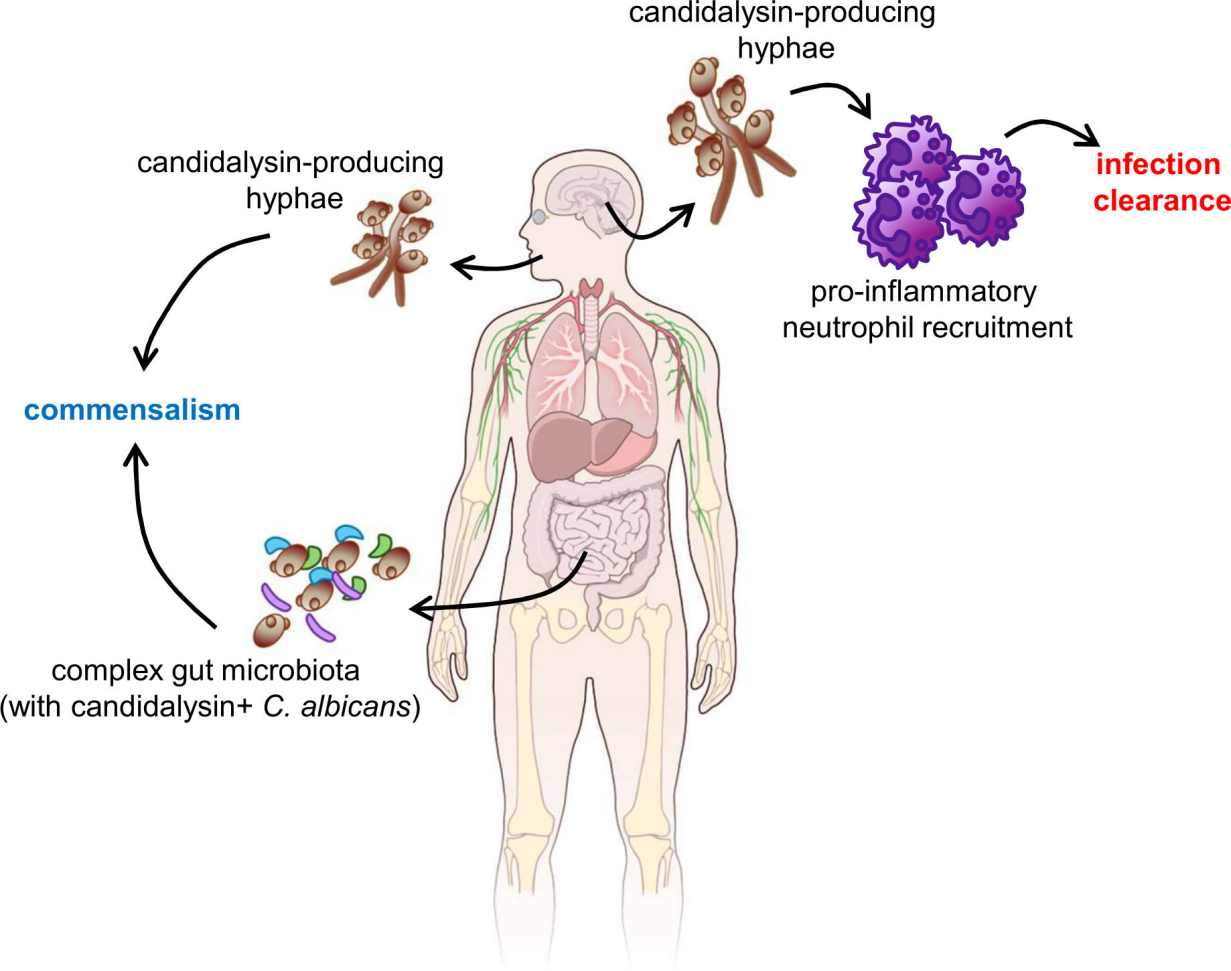
The multiple functions and roles of fungal toxin candidalysin. The various inflammatory and commensalism functions of *Candida albicans* toxin candidalysin are shown for different organs and infections to demonstrate the in-host adaptation that occurs for a fungal pathogen, which can be organ-specific and multifaceted. For example, in the brain, candidalysin drives pro-inflammatory neutrophil infiltration, which is required for fungal clearance and protective immunity. A similar response occurs at the oral mucosa when *C. albicans* invades the epithelial barrier. In low virulent strains with high commensalism potential, candidalysin is required to promote commensalism at the oral mucosa. A similar commensalism-promoting role is seen in the gut, particularly in the context of a complex microbiota. The human anatomy icon is from the NIH BioArt repository.

At the vaginal mucosa, the availability of zinc significantly influences the phenotype of *Candida albicans*, a common cause of fungal mucosal infections and responsible for an estimated 2 billion vaginal infections each year (11). When starved of zinc, *C. albicans* produces a zinc-scavenging molecule called Pra1, which is pro-inflammatory and stimulates neutrophil recruitment and responses (12). This inflammatory response to Pra1 drives symptoms of vaginitis. Restoring zinc levels at the vaginal mucosa in mice helped reduce neutrophilic inflammation, and small numbers of women who used a zinc-containing vaginal gel reported that their infections and symptoms were alleviated (12). For intracellular fungi, bioinformatic-based approaches helped reveal a significant expansion of cysteine-rich proteins called knottins in the fungus *Histoplasma capsulatum* (13). Knottins were found to be important for the fungus to survive within macrophage phagosomes and contributed toward virulence in mouse models of infection (13). Finally, the role of fungal virulence factors has been shown to differ depending on the environment the fungus is inhabiting and may therefore reveal niche-specific adaptation roles (Fig. 2). *C. albicans* produces a toxin called candidalysin, which was originally described as an important virulence factor during mucosal infections since candidalysin enabled fungal invasion across epithelial barriers (14). Since then, candidalysin has been shown to drive protective immune responses in other tissues and therefore contributed toward fungal clearance (15). In the case of commensalism, candidalysin is essential for maintaining *C. albicans* colonization in the gastrointestinal tract and at the oral mucosa (16, 17). Candidalysin expression appeared to be influenced by both host immunity and interactions with the gut microbiota, driving an intricate level of control that was necessary for fungal colonization and establishment of a growth niche in the host.

## IMMUNE METABOLISM AS A DRIVER OF INFECTION

How fungi adapt to their environment may also be influenced by metabolic challenges in immune cells, which have large energy demands to meet their functions. Recent years have seen more insights into how metabolism fuels immune responses to support antifungal immunity. We recently supported experiments that examined the phenotype of animals lacking NRK1, an enzyme involved with NAD synthesis, in the control of *C. neoformans* infection (18). We found that NRK1 was important for restraining T cell activation and function, since T cells lacking this enzyme produced greater levels of pro-inflammatory cytokines TNFα and IFNγ but exhibited greater levels of oxidative stress and cell death (18). During fungal infection, there were less effector T cells in infected organs, linked with increased expression of markers of oxidative damage and stress in T cells in NRK1-deficient animals, and a higher fungal burden in the brains of these mice (18). For other fungal pathogens, metabolic adaptation of immune cells is also crucial for infection outcome and patient susceptibility. For example, signaling via the C5ar1 receptor on macrophages was required to metabolically rewire these cells via mTOR and promote macrophage survival during *C. albicans* infection (19). C5ar1-deficient mice showed a loss of immunity to *C. albicans* associated with dysregulated macrophage metabolism and survival. Importantly, levels of complement C5 and polymorphisms in C5 were predictive of clinical outcome in patients (19). Metabolic abnormalities caused by diabetes, iron overload, or malnutrition have been found to predispose to infections with Mucorales fungi, which cause life-threatening and/or disfiguring infections (20). Recent work showed that a lack of the serum component albumin predicted worse clinical outcomes in patients with mucormycosis, which was linked with reduced production of fatty acids. The albumin-fatty acid pathway helped prevent the expression of fungal virulence factors and limited its growth in the host (20).

## THERAPIES BEYOND ANTIMICROBIAL DRUGS

Studying microbial in-host adaptation and the interplay between host metabolism and fungal virulence has led to many fascinating insights that will hopefully underpin alternative therapy approaches in the future. Immune-based therapies have been used very successfully in cancer treatment (21) and infections like severe COVID19 (22) but are under-explored in the context of other infections. Yet, these types of approaches can prove to be revolutionary, as was recently described in patients with autoimmune disorder autoimmune polyendocrinopathy-candidiasis-ectodermal dystrophy (APECED), who develop mucosal *C. albicans* infections as part of their condition (23). Mechanistic studies identified excessive production of IFNγ at the oral mucosa as a contributing factor toward susceptibility to fungal infection (24), and recent trials have shown that blocking the pathologic production of IFNγ reduced both the infection and autoimmune symptoms in APECED patients (25).

The future outlook for our lab and many others in the medical mycology field is to use these fundamental discoveries to develop new ideas for antifungal therapies, beyond antimicrobial drugs (26). Interdisciplinary collaboration is hugely important toward achieving this goal and has been critical in many of the new advances I have discussed here. I am excited to see more examples of microbiologists and immunologists coming together to tackle big questions in infectious disease research, which will lead to better management and control of invasive fungal infections, which still represent a significant and urgent threat to global human health.
